# Using an e-Health Intervention to Reduce Prolonged Sitting in UK Office Workers: A Randomised Acceptability and Feasibility Study

**DOI:** 10.3390/ijerph17238942

**Published:** 2020-12-01

**Authors:** Sophie E. Carter, Richard Draijer, Joseph D. Maxwell, Abigail S. Morris, Scott J. Pedersen, Lee E. F. Graves, Dick H. J. Thijssen, Nicola D. Hopkins

**Affiliations:** 1 Research Institute for Sport and Exercise Sciences, Liverpool John Moores University, Liverpool L3 3AF, UK; jmaxwell1@live.co.uk (J.D.M.); L.E.Graves@ljmu.ac.uk (L.E.F.G.); D.Thijssen@ljmu.ac.uk (D.H.J.T.); n.d.hopkins@ljmu.ac.uk (N.D.H.); 2 School of Science, Technology and Health, York St John University, York YO31 8TA, UK; 3 Unilever Foods Innovation Centre, Wageningen, The Netherlands; richard.draijer@unilever.com; 4 Department of Health Research, Lancaster University, Lancaster LA1 4YW, UK; a.morris7@lancaster.ac.uk; 5 Active Work Laboratory, School of Education, University of Tasmania, Launceston 7250, Australia; scott.pedersen@utas.edu.au; 6 Department of Physiology, Radboud Institute for Health Sciences, Radboud University Medical Center, PO Box 9101, 6500 HB Nijmegen, The Netherlands

**Keywords:** sedentary behaviour, workplace, prompts, cardiovascular health

## Abstract

Low-cost workplace interventions are required to reduce prolonged sitting in office workers as this may improve employees’ health and well-being. This study aimed to assess the acceptability and feasibility of an e-health intervention to reduce prolonged sitting among sedentary UK-based office workers. Secondary aims were to describe preliminary changes in employee health, mood and work productivity after using an e-health intervention. Healthy, university office workers (n = 14) completed this study. An 8 week randomised crossover design was used, consisting of two trials: Intervention (computer-based prompts) and Control. Eligibility and retention rates were recorded to assess the feasibility of the trial and interviews were conducted following the intervention to explore its acceptability. Sitting, standing and stepping were objectively assessed prior to and during week 8 of each trial. Before and after each trial, measurements of vascular function, cerebrovascular function, mood and work productivity were obtained. This study had eligibility and retention rates of 54.5% and 77.8%, respectively. Participants expressed a lack of autonomy and disruption to their workflow when using the e-health intervention, raising concerns over its acceptability and long-term implementation. Preliminary data indicate that the intervention may improve the patterning of activity accrued during work hours, with increases in the number of standing and stepping bouts completed, in addition to improving vascular function. This e-health intervention is feasible to deliver in a cohort of university office workers. However, adaptations to its implementation, such as personalised settings, are needed to increase acceptability before larger trials can be conducted.

## 1. Introduction

The workplace is where most employed adults accumulate high amounts of total and prolonged bouts of sedentary behaviour (SB), predominantly by sitting [1,2]. Since SB is an established independent risk factor for cardiovascular morbidity and mortality [3], workers are frequently exposing themselves to these potential health risks. Consequently, there is a need to change workplace activity patterns to facilitate regular breaks from sitting through increased standing and physical activity (PA) [4]. Nonetheless, there is little evidence from workplace intervention studies to support their efficacy in improving workers’ health [5].

A range of intervention strategies, including active workstations and sit-to-stand desks, have been employed to increase workplace PA and reduce sitting time, respectively [6]. Such interventions have shown promise, with systematic reviews reporting reductions in sitting time of 77–116 min per workday [6,7]. However, these strategies are limited by purchasing and installation costs, which may be beyond the financial budgets of some workplaces. As such, there is a need to examine alternative low-cost interventions [6]. One alternative strategy is using e-health interventions that deliver information electronically to individuals via computer and mobile technology [6]. In a recent review of technology enhanced interventions to reduce workplace SB, the most commonly used behavioural change techniques were prompts and cues [8]. This typically includes computer-based prompting software that provide messages to encourage workers to take breaks from prolonged sitting.

Interventions using computer-based prompting software have resulted in favourable changes in workplace behaviours, including daily increases in standing time [9,10,11] and reductions in sitting time and the number and duration of prolonged sitting bouts [12]. Some e-health interventions have, however, observed no changes in sitting time [13], possibly due to the strict automaticity of the hourly timed prompt, which did not restart once an activity break had been completed. This suggests that the design of some prompting software may not be suitable for all workers. It is therefore important to establish whether e-health interventions are acceptable and feasible among workers to enable appropriate adaptations to software to be made to increase their effectiveness. The primary aim of this study was therefore to assess the acceptability and feasibility of an e-health computer-based prompting software to promote a reduction in total and prolonged sitting time among sedentary UK-based office workers.

Reducing total and prolonged sitting bouts can benefit workers’ cardiometabolic health [14] and potentially enhance productivity and overall performance [4]. Interestingly, previous studies utilising e-health interventions have observed increases in workers’ self-report health and well-being [10], increased energy expenditure [11], and reductions in mean arterial pressure [9,15]. Whilst this approach therefore shows promise to improve workers’ health, these previous studies featured suboptimal study designs, relying on self-reported measures of health and energy expenditure [10,11] and lacking a control group [10,15]. There is therefore a need to explore the effect of using e-health interventions on markers of health and work performance using rigorous study designs. Understanding the possible implications on health and work performance, alongside acceptability and feasibility data, will provide important information regarding the efficacy of using an e-health intervention as a low-cost workplace intervention to reduce prolonged sitting. The secondary aims of this study were therefore to describe preliminary changes in sitting, standing and stepping time, markers of vascular function, cerebrovascular function, mood and work productivity after using an e-health intervention.

## 2. Materials and Methods

### 2.1. Participants

Office-based workers from one university (Liverpool John Moores University, Liverpool, UK) were recruited. Departmental managers were contacted to gain consent for employee recruitment (n = 49) and participation in this study, of which 17 approved. Staff within these departments were contacted via email with a study overview and those who expressed an interest received a participant information sheet and were screened for exclusion criteria including: part-time employment, aged >65 years, use of cardiometabolic medication, smoker, BMI >35 or <18 kg∙m^−2^, use of hormone-based contraception, pregnancy and diagnosis of cerebrovascular, cardiovascular or metabolic disease, or a mental health condition. Following this, 18 healthy office workers (7 male) were enrolled into this study and written informed consent was obtained prior to inclusion. Participants were given a voucher for £100 for their participation in this study.

### 2.2. Study Design and Procedures

**Figure 1:**
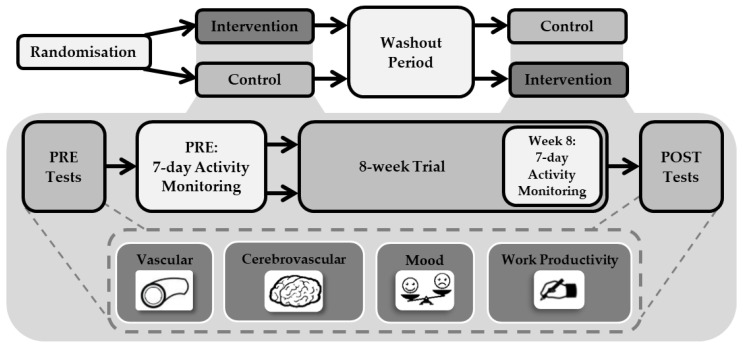
Study design.

Study procedures were approved by the Liverpool John Moores University Ethics Committee (17/SPS/034) and adhered to the Declaration of Helsinki. This study was conducted between September 2017 to May 2018. This study was a randomised crossover trial design, consisting of two trials: Intervention and Control. Each trial lasted 8 weeks, in line with previous research using this e-health intervention [16] and feasibility studies exploring interventions to reduce workplace sitting [17,18]. Trials were separated by a 6 week wash-out period. Participants were randomly assigned the order they completed trials by the principal researcher using computer-generated random numbers. During the Intervention trial participants used the e-health software to break up their workplace sitting, whilst in the Control trial participants did not have access to the software and were asked to maintain their normal workplace activity patterns. Participants and researchers were not blinded to group allocation. One week prior to commencing each trial (PRE), participants attended the laboratories at Liverpool John Moores University to complete measurements of vascular function, cerebrovascular function, mood and work productivity. Participants were then fitted with an activity monitor to measure their sitting, standing and stepping time, which was worn continuously for the next seven consecutive days. Immediately following this, the 8 week trial began. Sitting, standing and stepping time were assessed again during the final week of the trial (week 8). All other measures were then repeated directly after the 8 week trial (POST) (Figure 1).

### 2.3. e-Health Intervention

The e-health computer-based software, Exertime, was remotely installed onto the participant’s work computer the morning after the baseline activity monitoring period. Exertime is designed to prompt employees to interrupt prolonged sitting with brief bouts of PA during work hours [10,11] and is based on the habit formation theory [11,19]. The software provides more than 60 office-based PA options. However, since previous findings indicate beneficial cardiovascular effects from breaking up sitting with acute walking breaks [20,21], activity selection was limited to walking. To promote uptake of the software, on the morning that the software was remotely installed, participants were emailed an educational e-booklet about the importance of reducing their sitting time and instructions on how to initialise the software. Educational information has been used previously with the software [9,10,11] and increased its effectiveness in reducing prolonged sitting [16].

**Figure 2:**
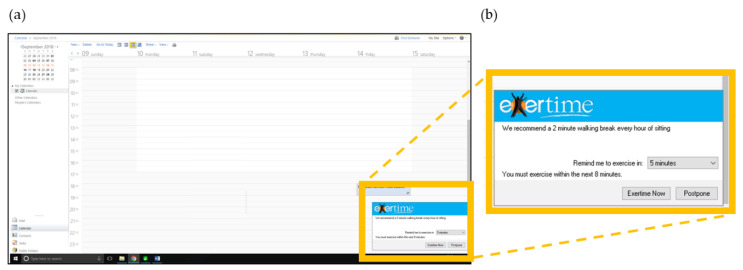
(**a**) Screenshot of the prompt at the bottom of the computer screen. (**b**) Close up of the prompt.

Once installed, the software was automatically initiated every 45 min as a prompt bubble appearing on the bottom right hand side of the participant’s computer screen, indicating it was time to take a break from sitting (Figure 2). The prompt frequency was based on previous studies using this intervention [10,11]. Participants could choose to either engage with or postpone the prompt. If engage was selected, the software displayed across the whole computer screen and could not be shut down, forcing the participants to click onto the software before being able to regain control of their computer screen. If postpone was selected, participants could temporarily delay the prompt for either 5, 10 or 15 min, for a maximum time of 15 min, after which the software was automatically activated. This function accounted for occasions where participants needed important access to their computer, such as a phone conversation or meeting. Participants were required to click the walking option to signify the beginning of their break and simultaneously initiate a timer. The break duration was self-selected. However, participants were advised via a message box presented on the screen to take a 2 min walking break. To end the break, the participant clicked to stop the timer, automatically logging the break duration in the software. The Exertime sequence then terminated and participants regained control over their computer screen. If a participant left their desk without the initiation of a prompt and did not lock their computer, the 45 min prompt timer was still active. The software could therefore activate without the participant being present. On these occasions, when participants returned to their desk, they could manually log the duration of time they had been active.

During the 8 week trial, use of the software was monitored by the principal researcher using the online portal for software administrators. If a participant was not logging activities, they were contacted via email by the principal researcher to check the software was working correctly (this occurred on one occasion due to computer updates deactivating the software). To increase motivation to engage with the software, participants received a weekly email from the principal researcher [9,22]. The email detailed the number of breaks and activity minutes they had logged in the previous week and provided a suggestion as to how they could break up their sitting with walking breaks (for example, ‘Going for a coffee break? Rather than sit with a colleague have a walking coffee break’). These suggestions varied each week but were the same for all participants throughout the intervention.

#### e-Health Software Data Analysis

Software usage data were recorded and accessible using the online administrator portal. For each participant, the date, time and duration of each break logged over the 8 week trial were downloaded and weekly averages for the number of breaks taken and the duration of each break calculated. The software usage data are based on the participants’ self-report activity and cannot be used to objectively verify participants’ fidelity to the intervention component. Consequently, to assess compliance to the breaks, objective monitoring data (activPAL) from the final week (week 8) of the Intervention trial were compared to software usage data from the same time period. Software usage data were time matched to the activPAL data files and each time a break was logged in the software, it was checked for a simultaneous break in the activPAL data file, allowing the compliance to software use (%) to be determined. The ‘sedentary to upright’ and ‘upright to sedentary’ data output from activPAL file indicates when a participant has transitioned from a sitting to standing posture or from a standing to sitting posture and therefore defined the start and end time of each break, respectively. If it was confirmed that a participant had taken a break, the accuracy of the logged break duration was verified by summing the epochs between these transitions to calculate the total activPAL break duration. If there were no activPAL transitions recorded during a logged software break, it was identified as a missed break. Participants were not aware their monitor data were being compared to their software usage data.

### 2.4. Primary Outcomes: Acceptability and Feasibility

Recruitment, eligibility and retention rates were recorded to assess the trial feasibility in addition to completion rates for the outcome measures. Calculations for recruitment (participants expressing interest in the study/participants enrolled × 100), eligibility (participants eligible/participants assessed for eligibility criteria × 100), and retention (participants completed the study/participants enrolled × 100) rates were performed, in addition to completion rates for all outcome measures (participants providing full outcome data/participants completing this study × 100). Participants were invited to attend a semi-structured interview following completion of the Intervention trial to elicit in-depth insights into the acceptability of the software. Discussion areas included participant experiences and perspectives of using the software, motivations for participation, frequency of breaks, reasons for using the delay function, perceived impact on health, mood and productivity outcomes, and factors influencing maintained use of the software during and beyond completion of the trial. A member of the research team experienced in qualitative research but not involved in intervention delivery (AM), randomisation or data collection developed the interview schedule, which was reviewed by members of the research team (SC, LG, NH). The protocol for delivery was standardised by using a semi-structured schedule to maintain a level of commonality across the interviews [23], while allowing flexibility in the order and sequence of questions to promote participants to respond openly and freely, using probes where appropriate to elicit depth from responses [24]. On the spot member checking was used to establish interpretation and meaning during interviews. Each interview was audio recorded, transcribed verbatim and anonymised during this process. Seven participants (n = 4 female) responded and took part, with the mean interview duration 27.9 ± 7.4 min.

### 2.5. Secondary Outcomes: Sitting, Standing and Stepping Time

For each assessment period, participants’ time spent sitting, standing and stepping were monitored continuously over five weekdays and two weekend days [25]. To delineate between work hours and leisure time behaviours, and to identify sleep, participants recorded the time they started and finished work and the time they woke up and went to bed each day in a logbook.

Sitting, standing and stepping time were assessed using a tri-axial activPAL monitor (PAL Technologies, Glasgow, UK) [26,27]. The activPAL was initialised at a sampling frequency of 20 Hz, waterproofed using a small flexible sleeve (PAL Technologies) and then secured onto the anterior mid-line of participants’ right upper thigh by the principal researcher using a waterproof medical grade adhesive dressing (Tegaderm, Bracknell, UK). Data were downloaded from the monitor using activPAL software (version 7.2.32) and saved in 15 s epochs across 24 h periods. Data for a day were invalid if the monitor was worn <10 h, had <500 steps recorded or any one activity accounted for ≥95% of waking wear time [28]. Visual inspection of the activPAL event file outputs corroborated if self-report wake-up and bedtimes were accurate. For inclusion, it was also required that the monitor was worn for >90% of work time and that participants had valid data for all measurement time points (PRE and POST) for both trials. Daily sitting, standing and stepping time, total step count and the number of sit-to-stand transitions were calculated for work hours. Additional analyses were performed using a validated algorithm [28] to further examine the effect of the e-health intervention by determining the number and total duration of sitting (0–30, 30–60, 60+ min), standing (0–30, 30+ min), stepping (0–30 min) and moderate-to-vigorous (MVPA) stepping (0–10, 10+ min) bouts during work hours. To account for variation in work time, data were normalised to an 8 h workday, as used previously [29,30].

### 2.6. Secondary Outcomes: Vascular Function, Cerebrovascular Function, Mood and Work Productivity

Prior to each laboratory visit, participants were instructed to avoid strenuous exercise for 24 h, abstain from alcohol and caffeine, and complete an overnight fast. Women were assessed in the follicular phase of the menstrual cycle (days 1–7). Anthropometric measures of stature and body mass were acquired at the start of each visit. After a 20 min supine rest, measures of vascular function and cerebrovascular function were obtained. Participants were then given a 15 min break and a standardised snack to consume. Following this, participants completed mood and work performance questionnaires.

#### 2.6.1. Anthropometry: Stature and Body Mass

Stature was measured to the nearest 0.1 cm using a portable stadiometer (SECA, Hamburg, Germany). In minimal clothing and without shoes, body mass was measured to the nearest 0.1 kg using an electronic scale (SECA 799, Hamburg, Germany). BMI was subsequently calculated (mass/stature^2^).

#### 2.6.2. Vascular Function

Resting heart rate (HR), systolic blood pressure (SBP) and diastolic blood pressure (DBP) and mean arterial pressure (MAP) were measured at the left brachial artery (Carescape V100, Dinamap, GE Healthcare, UK). Brachial and superficial femoral artery vascular function were assessed simultaneously using the non-invasive flow-mediated dilation (FMD) technique, which is a predictor of cardiovascular disease risk [31]. Assessments were conducted using high-resolution ultrasound (u-smart t3300; Terason, Burlington, MA, USA) according to the published guidelines [32] and are described in detailed elsewhere [33]. Briefly, after a 1 min of baseline, occlusion cuffs, connected to a rapid inflator (D.E. Hokanson, Bellevue, WA, USA), were inflated to 220 mmHg for 5 min. FMD was calculated as the absolute and percentage change in artery diameter from baseline to peak during the 3 min after cuff deflation. Data analysis was performed using custom-designed automatic edge-detection and wall-tracking software, as described in detail elsewhere [34].

#### 2.6.3. Cerebrovascular Function

Cerebrovascular function describes the regulatory mechanisms that maintain constant cerebral perfusion [35]. Acute impairments negatively influence cognitive functioning [36], whilst chronic alterations are associated with neurodegenerative disease risk [37,38]. Cerebrovascular function was assessed via resting cerebral blood flow (CBF), cerebral autoregulation (CA), cerebrovascular carbon dioxide (CO_2_) reactivity (CVR) and neurovascular coupling (NVC) [39]. Briefly, assessments were obtained at the temporal window using continuous bilateral transcranial Doppler ultrasound (TCD) (ST3, Spencer Technologies, Redmond, WA, USA) and are described in detail elsewhere [20].

Resting Cerebral Blood Flow. Supine middle cerebral artery blood flow velocity (MCAv) and posterior cerebral artery blood flow velocity (PCAv) were acquired as a 5 min average. The MCA accounts for 70–80% of the brain’s total perfusion, supplying the frontal, temporal and parietal brain regions whilst the PCA perfuses the occipital lobe [39].

Cerebrovascular CO_2_ Reactivity. Maintenance of adequate CBF is influenced by the brain’s ability to alter blood flow in response to changes in partial pressure of arterial CO_2_, termed CVR [35]. Testing procedures have been described in detail elsewhere [20], but briefly, after a 1 min baseline, participants voluntarily hyperventilated until the pressure of end tidal CO_2_ (PETCO_2_) was reduced to 20 mmHg. Participants then returned their respiratory rate to normal and inhaled a 5% CO_2_ mixture for 3 min. Simultaneously, to assess extracranial artery reactivity, arterial diameter and blood flow of the left common carotid artery (CCA) were measured. Absolute and relative MCAv and CCA diameter, and CCA blood flow reactivity to the changes in CO_2_ were calculated as previously described [20,40].

Cerebral Autoregulation. CA maintains adequate CBF over a range of perfusion pressures [35]. Participants completed two 5 min squat-stand tests to induce oscillations in BP [41]. Tests involved repeated cycles of 5 s of standing and 5 s of squatting (low frequency, 0.1 Hz) and 10 s of standing and 10 s of squatting (very low frequency, 0.05 Hz), separated with a 5 min rest. Data were processed and analysed in accordance with standardised transfer function analysis (TFA) guidelines to produce values of gain, phase and coherence [42]. These parameters are described in detail elsewhere [20].

Neurovascular Coupling. NVC measures temporal and regional CBF responses to neural activity [43] and is a significant determinant of cognitive performance [44]. Participants completed a visual stimulation task in accordance with published guidelines involving repeated cycles of eyes-open whilst viewing a visual stimulation screen, followed by eyes shut [43]. Data were analysed using automated software following recommended guidelines [43]. The absolute and percentage change in PCAv and MCAv from pre-visual stimulation were used to quantify the NVC response.

#### 2.6.4. Mood

Mood was assessed using The Positive and Negative Affect Schedule (PANAS; [45])). Participants were asked to respond using a 5-item Likert scale ranging from 1 (very slightly or not all all) to 5 (extremely) based on their mood over the past few days the extent to which they felt 10 positive and 10 negative states. Values were totalled to give separate positive and negative affect scores ranging from 10 to 50.

#### 2.6.5. Work Performance: Health and Work Questionnaire

Participants completed the Health and Work Questionnaire (HWQ; [46]) which is formed of 24 questions which then create subscales for: work productivity, concentration/focus, work satisfaction, non-work satisfaction, supervisor relations, impatience/irritability and stress. Participants were required to rate each item in the questionnaire on a ten-point scale, with the end points of the scale tailored to each specific question. Subscale scores were then derived by averaging items within a subscale. The HWQ significantly correlates to objective work performance [46].

### 2.7. Qualitative Analyses

Interview data were analysed using a thematic approach, which allowed the flexibility to identify themes across the complete data set in relation to the acceptability of the software [47,48]. Analysis began concurrently with data collection through a reflective commentary, which contained initial thoughts and emerging patterns in the early stages of analysis [49,50]. Transcriptions were read and re-read to familiarise the researcher with the complete data set, and initial codes were generated from a piece of text that related to factors influencing the acceptability of the software to promote breaks from sitting [50], prior to being imported into QSR NVivo software 10 package. During the inductive analysis process, higher-order themes were generated from emerging patterns within the initial coded data. Subthemes associated with higher-order themes were identified, providing a structure and a rich context. At this stage of analysis, the coding framework was reviewed by authors (SC, LG, NH) that allowed refinement of emerging themes [50], with this triangulation adding credibility and trustworthiness to the analysis process [49].

### 2.8. Data Analyses

There is no formal requirement to conduct a sample size calculation for feasibility studies [51,52]. Null hypothesis testing is not appropriate for pilot and feasibility studies and outcomes should be measured by descriptive statistics [53]. However, for exploratory purposes, preliminary trends in the data were explored by calculating effect sizes (Cohen’s *d*) of the between-group differences, achieved by dividing the difference in group means by the standard deviation of the pooled data. These were interpreted as: *d* = 0.2 considered small, *d* = 0.5 considered medium, and *d* = 0.8 considered large [54].

## 3. Results

### 3.1. Feasibility

**Figure 3:**
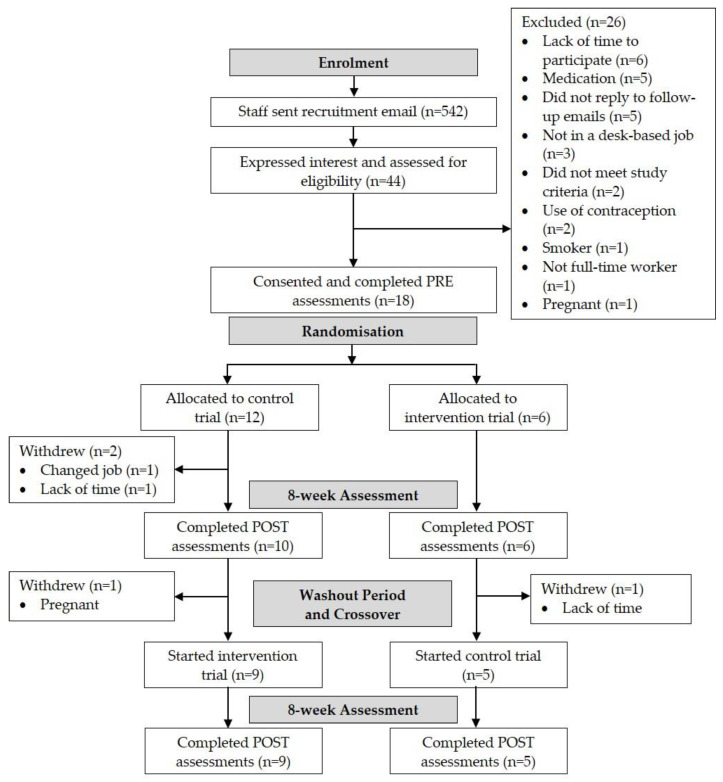
Consort flow diagram of enrolment, allocation and assessment.

Of the 44 participants who expressed interest, 11 declined to take part due to a lack of time to participate or failure to reply to follow-up emails. From the remaining 33 participants, 15 were not eligible, meaning 18 participants were enrolled onto this study (eligibility rate = 55%; recruitment rate = 41%). From the originally recruited sample size of 18, 14 participants completed this study (retention rate = 78%). Two participants withdrew after initial allocation to the control trial, one due to a change of job and one due to lack of time. Two participants withdrew during the washout period, one due to pregnancy and one due to lack of time (Figure 3). Full descriptive characteristics are shown in Table 1. The 14 participants that completed this study provided 100% of data for sitting, standing and stepping time, vascular function, mood and work productivity. For cerebrovascular function data, signal acquisition was not possible for one participant, meaning 13 (93%) participants provided data for these outcomes. Seven participants completed the semi-structured interviews (50%).

### 3.2. Acceptability

From the qualitative interviews, participants perceived and experienced the software in a heterogeneous manner, with four broad themes emerging from the interview data: perceptions of the software; types of break modalities; impact on health, well-being and work-related outcomes; and maintenance of behaviour (Table 2). Perceptions of the software encompassed a range of positive and negative factors that influenced participants’ perceptions regarding its acceptability. There was a consensus among participants that the prompting feature provided a useful cue to break up prolonged periods of sitting at work. Some participants reported that the break warning allowed them to structure their daily workload and mentally prepare working tasks in order to adhere to a break. Most participants felt they would prefer a less intrusive prompt (whereby they can voluntarily initiate the software by clicking on the icon), compared to the ‘*intrusive’, ‘aggressive’, ‘frustrating’* and *‘annoying*’ lock-out feature of the software. However, some also identified that this type of prompt may be less effective for promoting adherence to breaks. Overall, participants disliked the lack of autonomy over the lock out feature of the software and the type of break available throughout the Intervention trial (which was limited to walking only). Collectively, participants found the software ‘*clunky*’ and that it lacked sensitivity for detecting appropriate break times accurately as it would assume that participants were sedentary even if they were away from their desk (i.e., if a prompt was missed).

Despite being advised to break up their sitting with walking breaks, participants described adopting a range of modalities during their break consisting of purposeful activities to increase PA (i.e., stair use, squats and lunchtime walks), and activities to increase incidental PA integrated into working patterns (i.e., photocopying, talking to colleagues and tea breaks). The type of break appeared to depend on the time available and job demands when the software prompt was activated throughout the day, as well as participants’ health driven motivation to take part in the intervention. The impact of the intervention on work-related outcomes included some disruption to workflow and concentration and some participants often found it was inconvenient during meetings, one-to-ones and project work. Subsequently, some participants perceived increased stress, anxiety and frustration during the intervention period associated with use of the software. Conversely, enforced breaks due to the lock out feature also encouraged participants to take a physical and mental break away from work, which reportedly had some positive impact on stress, fatigue, productivity and physical fitness.

Overall participants reported that they would not continue to use the software in its current format if made available by their employer. Despite most participants reporting an increased awareness of sitting and health at work since termination of the intervention, participants reported that their break frequency had reduced and typically relied on other cues such as mood, workload and use of facilities, to prompt breaks to sitting. Some participants reported that they continued to integrate PA into breaks such as taking a walk during lunch times following the trial.

### 3.3. e-Health Software Usage

**Figure 4:**
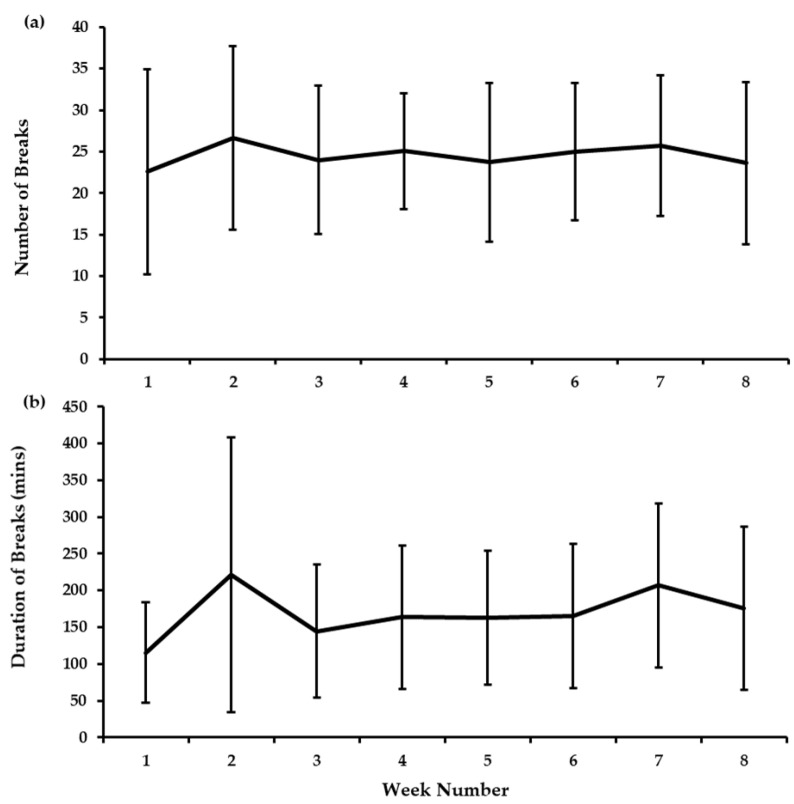
Weekly e-health software data for (**a**) the number of breaks recorded, and (**b**) the total duration of the breaks (mean ± SD).

Week-by-week data for the number and duration of breaks recorded by participants from the automated software are shown in Figure 4. Over the 8 week intervention, the automated software recorded a daily average of 8.0 ± 3.1 min of breaks from sitting, as assessed by standing or stepping time, achieved over 5.8 ± 1.2 breaks per day. This equated to 172.4 ± 67.4 min taking breaks per week, over 24.7 ± 6.5 breaks. The corresponding activPAL data from week 8 of the intervention indicated that participants took a break from sitting for 68.0% of the breaks that were logged. Of these breaks with corresponding objective activPAL data, the break duration recorded by the software (108.8 ± 83.9 min/week) was higher than that recorded by the activPAL (97.2 ± 70.6 min/week, *d* = 0.16).

### 3.4. Sitting, Standing and Stepping Time

The time spent sitting, standing and stepping during work hours are presented in Table 3. Large effects were observed for the change in total minutes (*d* = 0.92) and the percentage of work hours (*d* = 0.89) spent sitting in favour of Intervention. Large effects were also observed for the change in total minutes (*d* = 0.88) and the percentage of work hours (*d* = 0.87) spent standing in favour of Intervention. The effect sizes for all other outcomes were small. The number and total duration of sitting, standing and stepping bouts during work hours are presented in Table 4. There were large effects for the change in the number of standing bouts lasting 0–30 min (*d* = 0.84) and the number (*d* = 0.99) and duration (*d* = 1.40) of standing bouts lasting >30 min in favour of Intervention. Large effects were also observed for the change in the number of stepping bouts lasting 0–30 min (*d* = 1.08) and the number of MVPA stepping bouts lasting 0–10 min (*d* = 1.13) in favour of Intervention. The effect sizes for all other outcomes were medium or small.

### 3.5. Vascular Function

Data for resting HR and BP are presented in Table S1. There were large effects observed for the change in SBP (*d* = 0.84) and MAP (*d* = 1.02) in favour of Control. The effect sizes for HR and DBP were small. Data for brachial and femoral artery FMD are presented in Table 5. Large effects were observed for the change in absolute (*d* = 0.88) and relative (*d* = 1.06) femoral artery FMD in favour of Intervention. The effect sizes for all other outcomes were small. The effect sizes for all brachial artery FMD outcomes were medium or small.

### 3.6. Cerebrovascular Function

Data for resting CBF are presented in Table S1. The effect sizes for all CBF outcomes were small. Data for CA, CVR and NVC are presented in Table S2. For CA, in the LF squats (5 s squat, 5 s stand) large effects were observed for the change in gain (*d* = 1.25) and normalised gain (*d* = 0.91) in favour of Control. Effect sizes for all other CA outcomes, and for all NVC and CVR outcomes were medium or small.

### 3.7. Mood and Work Productivity

Mood and work productivity data are presented in Table S3. Effect sizes for all measures of mood and work productivity were medium or small.

## 4. Discussion

This study assessed the acceptability and feasibility of implementing an e-health prompting software to promote a reduction in total and prolonged sitting time among university-based UK office workers. Secondary aims were to describe preliminary changes in sitting, standing and stepping time, in addition to aspects of health, mood and work productivity after using the software. Firstly, the trial was feasible to deliver with this cohort, with a low dropout rate, and successful collection of outcome variable data. However, qualitative findings from participant interviews suggest that this method of implementing the e-health software may not be an acceptable strategy in university office workers. Indeed, a lack of autonomy when using the software and disruption to workflow may compromise its use in the long term with this cohort. This indicates adaptations to the prompting feature of the software may be required and should be subsequently explored in future research. Nonetheless, our preliminary data indicate that the e-health software may improve the patterning of activity accrued during work hours, with increases in the number of standing and stepping bouts participants completed, and may also improve femoral artery function, a marker of cardiovascular health.

The trial was feasible to implement in this population of university-based office workers. The retention rate during this study was 78%, with reasons for participant withdrawal due to personal reasons (e.g., pregnancy) or lack of time associated with the pre and post testing procedures, rather than the e-health intervention itself. Furthermore, over the 8 week duration, only one participant experienced an issue with the software, which occurred on one occasion. The eligibility rate was modest (55%); likely in part due to our exclusion of participants with cerebrovascular, cardiovascular or metabolic diseases, or a mental health condition. Unpublished data from our laboratory indicate that expanding eligibility criteria to include cardiometabolic conditions increases response rate by 13%; thus, future trials should consider this. Compliance with providing outcome measure data was high, with participants providing 100% of data for all outcomes except cerebrovascular function (93%) and semi-structured interviews (50%). The lower completion percentage for the semi-structured interviews may reflect the time of data collection, which occurred at the end of the Intervention trial and coincided with winter and summer vacation periods at the University; thus, participants were often taking annual leave and unable to partake.

Despite our feasibility data, qualitative insights from participants, combined with the objective assessment of compliance to the software, suggest this method of implementation may not be an acceptable strategy for the long-term use of this e-health intervention. Indeed, some participants reported they would not continue to use the software in its current form if it was made available to them by their employer. Furthermore, participants indicated that the software may influence, both positively and negatively, their mood and work productivity. Some participants found the prompts disrupted their workflow and were inconvenient during specific tasks such as meetings, which led them to describe feelings of stress and frustration whilst using the software. Indeed, a loss of productivity when taking activity breaks from sitting is a commonly reported concern from employees [55]. Furthermore, pressurised work tasks and losing track of time have been described as barriers to taking activity breaks when using a prompting software to reduce workplace sitting [56]. Alternatively, other participants reported that the break provided a mental break from their work, reducing their fatigue and stress. This latter finding aligns with previous research using the software whereby participants’ self-report health and well-being increased following the intervention period [10]. Additionally, qualitative insights from participants using an alternative prompt-based e-health intervention have reported feeling refreshed after taking an activity break from sitting [56]. Despite these qualitative findings, the preliminary findings from our quantitative measures of mood and work productivity showed no changes, although findings should be interpreted with caution as they likely lack statistical power. Some participants also highlighted concerns over the sensitivity of the software to accurately reflect activities they completed when they were away from their desk. Collectively these data suggest adaptations to the software are required to make it suitable for the individual needs of a workforce, for example personalised settings for different job roles. The alterations to the software that workers feel are necessary should be explored further, and co-developed with appropriate office-based workers, researchers, and software developers.

The software recorded that participants logged an average eight additional minutes taking breaks from sitting, achieved across six breaks per workday, which is comparable to previous studies using the software as a workplace intervention [9,10,11]. However, these studies only relied on the data from the software’s online portal, which is not able to discern whether participants actually took a break. In this study, for the first time, the objective activity monitoring provided an initial assessment of the validity of the software by comparing participants’ self-reported breaks to objective data. Using this approach, it was observed that participants completed 68% of the breaks they logged in the software. This level of adherence to the prompts may reflect participants’ dissatisfaction with the timed prompting feature interrupting them during work events and the desire for it to be less intrusive whereby they can voluntarily initiate the software. Indeed, participants wanted greater autonomy over the lock out feature of the software and the type of break they could choose. Frustrations from the prompts may therefore have meant participants disengaged or ignored some prompts over the course of their workday. Similarly, an alternative e-health intervention using automatic hourly timed prompts observed no change in workplace sitting time [13], while an e-health intervention using randomly embedded prompts into employees’ Microsoft Outlook calendars reported that participants ignored the prompts before the end of the intervention period [56]. Collectively these findings indicate prompts should be tailored to the individual behaviour of each worker. Future research employing this e-health intervention should consider a more sensitive prompt that does not solely rely on a timer, for example prompts that are synchronised to work events.

The preliminary analyses of the objective behavioural data indicate that the intervention may alter the activity accrued during work hours. Following the intervention, total workplace sitting time decreased by 12 min (2.4%) whilst total standing and stepping time increased by 11 min (2.3%) and 1 min (0.1%), respectively. This reduction in sitting time is similar to an alternative e-health intervention which reduced total workplace sitting by 14 min [6], but is modest compared to reductions of 77–116 min when using active or sit-to-stand workstations [6,7]. These interventions allow workers to engage in activity whilst remaining at their desk, whereas the software used in this study promoted walking breaks away from the desk, which may explain these differences. However, despite advising participants to complete a walking break, it is interesting that the greatest change in behaviour was derived from standing, indicating participants may prefer more choice in the activities they complete during their breaks, which was reflected in the qualitative data. The intervention may also positively change the pattern in which behaviour is accrued during work hours, as the number of stepping bouts (0–30 min bouts and 0–10 min MVPA bouts) and the number and total time spent in standing bouts lasting 30+ min increased. However, there were minimal changes in the number of sit-to-stand transitions, which has also been observed when using an alternative prompt-based workplace intervention [13]. This may indicate participants did not take more breaks from sitting but perhaps instead did an activity for longer or completed consecutive activities during each of these breaks, for example walking further or walking to a colleague to have a standing conversation. Thus, for some participants, the software prompts may have just served as a way of scheduling their normal breaks from sitting to carry out incidental workplace activities, such as toilet breaks, or purposeful activities, such as walking during lunch breaks, and they spent more time engaged in these activities. Consequently, whether this method of implementing the e-health intervention is effective at increasing the daily number of breaks from sitting workers complete is unclear and further research is needed to corroborate these preliminary findings.

The preliminary effects of the e-health software on measures of vascular function and cerebrovascular function were also assessed. Using the software may positively influence vascular function as following the intervention femoral artery FMD increased by 4.8%. Importantly, FMD is a surrogate marker for future cardiovascular events [31] indicating that, if the observed software usage was continued over a longer duration, it could have important implications for the prevention of and reduction in cardiovascular disease risk in sedentary workers. Although cerebrovascular function did not improve following the intervention, this is the first time these measures have been assessed specifically in office workers, and importantly they were feasible to complete (93% completion rate). Consequently, future research should continue to explore whether interventions designed to alter workplace activity can influence cerebrovascular function.

### Strengths and Limitations

The main strength of this study was the evaluation of the acceptability and feasibility of an e-health intervention utilising both qualitative participant insights and objective measures of sitting, standing and stepping time, vascular function, and cerebrovascular function. Importantly, the objective assessment of behavioural outcomes minimises the risk of reporting or recall bias [57]. This study’s findings can be used to adapt the prompting feature of the software with the aim of increasingly its acceptability and effectiveness, and this can be assessed in future trials. The limitations of this study include the small sample size and therefore preliminary analyses that were conducted. Our recruitment rate of 41% could have been improved by using strategies such as team leader/management buy-in and support, as has been used in previous workplace intervention studies [18], thus future trials should consider this approach. The population assessed were all employees from a university, meaning software usage, compliance and acceptability may differ in other worksites and professions. Despite this, our sample’s workplace sitting behaviours are representative of other workforces. At baseline, workers spent 318 min (Control PRE) and 345 min (Intervention PRE) sitting whilst at work. This is comparable to a recently published systematic review and meta-analysis of device-measured sitting at work that reported workers from all occupations spend 312 min of their workday sitting, while specifically office workers spend 340 min of their workday sitting [58]. Only healthy adults were recruited, meaning results may differ in clinical populations, future studies should seek to include those with health-related risk factors and health conditions who would likely benefit most from health interventions. Acceptability and feasibility could have been explored further through quantitative survey-based methods. Furthermore, qualitative data surrounding the acceptability of outcome measures could have been collected. For the purpose of this study, participants’ activity selection when using the software was limited to taking a walk, whereas full use of the software includes a range of activity modalities. The lack of activity choice may have reduced participant engagement with the software, thus future research could replicate this design but provide participants the full range of activity choices. It is also possible that seasonality may have influenced participants’ activity levels, since these can vary depending on the season when the assessment occurs [59]. Similar to previous feasibility studies [17,18], the intervention was conducted over eight weeks, therefore a longer intervention period and follow ups are needed explore the sustainability and effectiveness of this e-health intervention and also whether the intervention results in long-term habit gains.

## 5. Conclusions

This study demonstrates that implementing an e-health prompting software for eight weeks and collecting objective outcome measures including sitting, standing and stepping time, vascular function, and cerebrovascular function is a feasible intervention for a cohort of university office workers. However, due to lack of autonomy in the prompting feature, participants indicated that the software was generally not acceptable in its current form. Despite this, preliminary data indicated that using the software may increase the number of standing and stepping bouts during work hours and improve a marker of cardiovascular health. Consequently, whilst this workplace e-health intervention shows promise, adaptations to the software are needed in order to improve acceptability. Following these adaptations, research should further explore the software’s acceptability, before larger trials are conducted.

## Figures and Tables

**Table 1:** Participant descriptive characteristics (n = 14, 6 male).

	Mean ± SD or n(%) of Group
Age (years)	42.5 ± 10.0
Body Mass (kg)	76.8 ± 19.5
Stature (cm)	169.9 ± 9.5
Body Mass Index (kg∙m^−2^)	26.3 ± 4.4
White British	14 (100)
Married	10 (71)
**Job Category**	
Clerical	3 (21)
IT Services	4 (29)
Research and Development	2 (14)
Teaching Services/Support	5 (36)
**Time at Current Workplace**	
<1 year	1 (7)
1–3 years	5 (36)
>3 years	8 (57)
Work Hours (per week)	37 ± 8
Work Hours (per day)	8 ± 1
**Number of People in Office**	
0	2 (14)
1–3 People	3 (21)
>3 People	9 (64)
**Occupational Transport**	
Car	5 (36)
Train	5 (36)
Bus	3 (21)
Walk	1 (7)

**Table 2:** Participant perceptions of the acceptability of the e-health software.

Perceptions of the software (a) Mental preparation (b) Passive prompts (c) Lack of autonomy (d) Sensitivity of software	(a) ‘I’d be working away, “Oh, I’m not quite ready for a break”, and the extra five minutes, I think it starts like mentally preparing you, so you start winding things up a bit to a point where you can finish, so that is really useful.’ p. 12. (b) ‘[The software] was aggressive, but I think it actually means it’s effective, whereas [prompts alone] isn’t quite so effective’ p. 12. (c) ‘Giving you the option or the choice to do something would be better’ p. 10. (d) ‘[The software] didn’t recognise perhaps when you’d gone off to teach. So I would come back in, and you’d almost have to falsify the information, because it’d be saying, “What have you done?” But you might have walked to three different […] buildings, and actually not been stagnant, but it didn’t recognise, it just thought, you’re not at your computer. It always thought you were sat stagnant, so you need to get up and move. […] It probably didn’t give the truest reflection […] Sometimes you’d come back and you just put, I don’t know, two hours, but you weren’t walking for two hours. It was difficult to know.’ p. 15.
Types of break modalities (a) Purposeful PA (b) Incidental PA integrated into working tasks	(a) ‘I’d just walk up and down the stairs […] It’s two flights. I used to do that twice, and that’d be just over five minutes.’ p. 6. (b) ‘It was a prompt to get up and move, and rather than perhaps sending all your photocopying at the end of the day […] I’d probably go throughout the day, so I could utilise the [software] breaks, then I’ll go, and it was just a good way to get up and move, really.’ p. 15.
Impact on; (a) Health, well-being; (b) Work-related outcomes	(a) ‘I did feel better. Just a bit of calm, bit of peace and quiet […] so it’s quite nice just to clear your head, and like I say, just get a bit of fresh air and stretch your legs.’ p. 13. (b) ‘If I inadvertently was typing, and it [the software] came up, and I hit the carriage return or something, it became the active window, so therefore it launched straight away, rather than just sitting there […] I didn’t lose any work, it was just, frustrating.’ p. 3.
Maintenance of behaviour (a) Reduced frequency of breaks (b) Incorporating PA into breaks	(a) ‘Now the software’s disabled on my pc, it could be an hour and a half before I actually get up to take the break sort of thing, but I obviously will get up at some point, and obviously go and make myself and my colleague a drink or whatever, or I don’t tend to walk up and down the stairs now, but sometimes a trip to the loo or it’s a trip to the kitchen, that sort of thing.’ p. 3. (b) ‘[Since completing the trial] I still go out every day, so that’s good.’ p. 12.

**Table 3:** Time spent sitting, standing and stepping during work hours at the start (PRE) and during week 8 of the Control and Intervention trials (mean ± SD).

	Control		Within-Group Differences	Intervention		Within-Group Differences	Between-Group Differences	Cohen’s d
	PRE	Week 8		PRE	Week 8			
Sitting Time (min/8 h workday)	318.3 ± 66.8	344.7 ± 43.2	26.3 ± 43.7	345.0 ± 37.6	333.1 ± 57.5	−11.9 ± 43.2	−38.2 ± 72.9	0.92
Standing Time (min/8 h workday)	108.4 ± 64.2	83.2 ± 34.7	−24.6 ± 45.5	82.3 ± 36.0	93.5 ± 41.2	11.2 ± 38.5	35.8 ± 69.2	0.88
Stepping Time (min/8 h workday)	53.2 ± 14.8	51.5 ± 18.5	−1.7 ± 16.5	52.7 ± 17.5	53.4 ± 24.6	0.7 ± 21.5	2.4 ± 22.4	0.13
Sitting Time (% of work hours)	66.6 ± 13.7	71.9 ± 8.6	5.3 ± 8.6	72.0 ± 7.8	69.6 ± 12.0	−2.4 ± 9.4	−7.7 ± 15.0	0.89
Standing Time (% of work hours)	22.4 ± 13.1	17.4 ± 7.0	−4.9 ± 9.1	17.1 ± 7.4	19.4 ± 8.6	2.3 ± 8.2	7.2 ± 14.3	0.87
Stepping Time (% of work hours)	11.0 ± 3.1	10.6 ± 3.8	−0.4 ± 3.3	10.9 ± 3.7	11.0 ± 5.1	0.1 ± 4.5	0.5 ± 4.7	0.13
Sit-to-Stand Transitions (n/8 h workday)	26 ± 8	24 ± 5	−2 ± 5	26 ± 8	25 ± 5	−1 ± 7	1 ± 7	0.17
Step Count (n/8 h workday)	5156 ± 1554	5176 ± 2039	20 ± 1725	5205 ± 1719	5149 ± 2646	−56 ± 2316	−76 ± 2333	0.04

**Table 4:** The number and duration of sitting, standing and stepping bouts during work hours at the start (PRE) and during week 8 of the Control and Intervention trials (mean ± SD).

	Control		Within-Group Differences	Intervention		Within-Group Differences	Between-Group Differences	Cohen’s *d*
	PRE	Week 8		PRE	Week 8			
Sitting Bouts								
0–30 min (n/8 h workday)	22.4 ± 9.3	21.4 ± 6.3	−1.0 ± 5.3	23.5 ± 9.5	21.3 ± 6.0	−2.2 ± 7.3	−1.2 ± 6.5	0.20
30–60 min (n/8 h workday)	2.6 ± 1.2	2.7 ± 1.1	0.1 ± 1.3	2.2 ± 1.1	2.8 ± 1.4	0.6 ± 1.9	0.5 ± 2.4	0.32
60+ min (n/8 h workday)	0.8 ± 0.9	0.9 ± 0.8	0.1 ± 0.8	1.0 ± 0.9	0.8 ± 0.6	−0.2 ± 0.8	−0.3 ± 1.0	0.39
Total Time 0–30 min (hrs/8 h workday)	2.5 ± 0.7	2.7 ± 0.8	0.2 ± 0.6	2.8 ± 1.0	2.6 ± 0.7	−0.2 ± 0.8	−0.4 ± 0.8	0.59
Total Time 30–60 min (hrs/8 h workday)	1.8 ± 0.8	1.9 ± 0.8	0.1 ± 1.0	1.6 ± 0.8	1.9 ± 0.9	0.3 ± 1.2	0.2 ± 1.6	0.19
Total Time 60+ min (hrs/8 h workday)	1.1 ± 1.3	1.2 ± 1.0	0.1 ± 1.1	1.3 ± 1.3	1.0 ± 0.8	−0.3 ± 1.2	−0.4 ± 1.5	0.18
Standing Bouts								
0–30 min (n/8 h workday)	148.3 ± 54.8	139.9 ± 58.3	−8.4 ± 31.9	138.3 ± 58.2	152.8 ± 64.2	14.5 ± 24.1	22.9 ± 50.8	0.84
30+ min (n/8 h workday)	0.2 ± 0.5	0.0 ± 0.1	−0.2 ± 0.4	0.0 ± 0.0	0.1 ± 0.2	0.1 ± 0.2	0.3 ± 0.6	0.99
Total Time 0–30 min (hrs/8 h workday)	1.7 ± 0.8	1.4 ± 0.6	−0.3 ± 0.5	1.5 ± 0.7	1.5 ± 0.6	0.0 ± 0.7	0.3 ± 0.9	0.51
Total Time 30+ min (hrs/8 h workday)	0.2 ± 0.4	0.0 ± 0.1	−0.2 ± 0.3	0.0 ± 0.0	0.1 ± 0.1	0.1 ± 0.1	0.3 ± 0.4	1.40
Stepping Bouts								
0–30 min (n/8 h workday)	172.0 ± 67.6	157.1 ± 71.0	−14.8 ± 40.8	161.6 ± 71.9	185.6 ± 94.7	24.0 ± 33.3	38.8 ± 51.2	1.08
MVPA 0–10 min (n/8 h workday)	195.1 ± 70.1	181.2 ± 82.6	−13.9 ± 46.6	181.4 ± 77.6	209.8 ± 103.2	28.4 ± 29.4	42.3 ± 49.5	1.13
MVPA 10+ min (n/8 h workday)	0.1 ± 0.1	0.1 ± 0.2	0.0 ± 0.1	0.1 ± 0.3	0.0 ± 0.1	−0.1 ± 0.3	−0.1 ± 0.4	0.47
Total Time 0–30 min (hrs/8 h workday)	0.2 ± 0.1	0.2 ± 0.1	0.0 ± 0.0	0.2 ± 0.1	0.2 ± 0.1	0.0 ± 0.1	0.0 ± 0.1	0.00
Total Time MVPA 0–10 min (hrs/8 h workday)	0.6 ± 0.2	0.7 ± 0.3	0.1 ± 0.2	0.7 ± 0.2	0.7 ± 0.3	0.0 ± 0.3	−0.1 ± 0.3	0.41
Total Time MVPA 10+ min (hrs/8 h workday)	0.0 ± 0.0	0.0 ± 0.0	0.0 ± 0.0	0.0 ± 0.1	0.0 ± 0.0	0.0 ± 0.1	0.0 ± 0.1	0.00

MVPA—moderate-to-vigorous physical activity.

**Table 5:** Brachial and femoral artery flow-mediated dilation (FMD) at the start (PRE) and following (POST) the 8 week Control and Intervention trials (mean ± SD).

	Control		Within- Group Differences	Intervention		Within- Group Differences	Between- Group Differences	Cohen’s *d*
	PRE	POST		PRE	POST			
Brachial Artery								
Baseline Diameter (cm)	0.34 ± 0.09	0.34 ± 0.09	0.00 ± 0.02	0.36 ± 0.08	0.35 ± 0.09	−0.01 ± 0.02	−0.01 ± 0.03	0.52
FMD (%)	6.1 ± 3.6	7.3 ± 4.3	1.2 ± 3.4	6.5 ± 3.9	7.9 ± 4.5	1.4 ± 5.8	0.2 ± 6.2	0.04
Absolute FMD (cm)	0.02 ± 0.01	0.02 ± 0.01	0.00 ± 0.01	0.02 ± 0.01	0.03 ± 0.01	0.01 ± 0.02	0.01 ± 0.02	0.66
SR AUC (s^−1^ × 10^3^)	23.58 ± 12.34	26.09 ± 9.06	2.50 ± 10.95	27.15 ± 16.07	26.18 ± 12.20	−0.97 ± 18.86	−3.48 ± 19.89	0.23
Femoral Artery								
Baseline Diameter (cm)	0.63 ± 0.15	0.61 ± 0.12	−0.02 ± 0.05	0.62 ± 0.14	0.61 ± 0.15	−0.01 ± 0.03	0.01 ± 0.06	0.25
FMD (%)	6.5 ± 2.7	5.9 ± 4.3	−0.6 ± 4.4	5.3 ± 3.2	10.5 ± 6.3	5.2 ± 6.7	5.8 ± 6.9	1.06
Absolute FMD (cm)	0.04 ± 0.01	0.04 ± 0.02	0.00 ± 0.03	0.03 ± 0.02	0.06 ± 0.03	0.03 ± 0.04	0.03 ± 0.05	0.88
SR AUC (s^−1^ × 10^3^)	19.27 ± 10.89	16.86 ± 5.89	−2.41 ± 9.88	19.21 ± 12.26	20.16 ± 11.08	0.95 ± 12.42	3.37 ± 12.70	0.31

SR—shear rate; AUC—area under the curve.

## Supplementary Materials

The following are available online at https://www.mdpi.com/1660-4601/17/23/8942/s1, Table S1: Resting cardiovascular and cerebrovascular measures at the start (PRE) and following (POST) the 8 week Control and Intervention trials (mean ± SD); Table S2: Measures of cerebrovascular function at the start (PRE) and following (POST) the 8 week Control and Intervention trials (mean ± SD); Table S3: Measures of mood and work productivity at the start (PRE) and following (POST) the 8 week Control and Intervention trials (mean ± SD).
