# Supplementary material for: Using an e-Health Intervention to Reduce Prolonged Sitting in UK Office Workers: A Randomised Acceptability and Feasibility Study
Source: Int J Environ Res Public Health. 2020 Dec 1;17(23):8942. doi: 10.3390/ijerph17238942 (PMC7729470; doi:10.3390/ijerph17238942)
Supplement: Supplementary file 1 [file ijerph-17-08942-s001.pdf]

Table S1. Resting cardiovascular and cerebrovascular measures at the start (PRE) and following (POST) the 8 week Control and Intervention trials (mean ± SD).

	Control		Within-Group	Intervention		Within-Group	Between-Group	Cohen's *d*
	PRE	POST	Differences	PRE	POST	Differences	Differences	
**Cardiovascular**								
HR (bpm)	62 ± 10	62 ± 12	0 ± 5	58 ± 11	57 ± 11	-1 ± 7	-1 ± 8	0.17
SBP (mmHg)	125 ± 14	121 ± 15	-4 ± 5	120 ± 11	122 ± 14	2 ± 8	6 ± 11	0.93
DBP (mmHg)	77 ± 9	75 ± 10	-2 ± 6	73 ± 10	74 ± 8	1 ± 7	3 ± 9	0.48
MAP (mmHg)	93 ± 10	90 ± 11	-3 ± 4	88 ± 9	90 ± 10	2 ± 6	5 ± 6	1.02
**Cerebrovascular**								
MCAv ($cm \cdot s^{-1}$)	70.1 ± 12.0	68.3 ± 8.4	-1.8 ± 7.0	67.4 ± 14.1	66.4 ± 9.1	-1.0 ± 8.3	0.8 ± 11.1	0.11
PCAv ($cm \cdot s^{-1}$)	52.4 ± 13.3	51.3 ± 9.8	-1.1 ± 7.8	48.9 ± 9.1	50.2 ± 10.9	1.3 ± 9.1	2.4 ± 13.7	0.30
$PETCO_2$ (mmHg)	38 ± 4	39 ± 4	1 ± 2	36 ± 5	37 ± 5	1 ± 4	0 ± 5	0.00

HR—heart rate, SBP—systolic blood pressure; DBP—diastolic blood pressure, MAP—mean arterial pressure, MCAv—middle cerebral artery blood flow velocity, PCAv-—posterior middle cerebral artery blood flow velocity, $PETCO_2$—pressure of end-tidal carbon dioxide.

Table S2. Measures of cerebrovascular function at the start (PRE) and following (POST) the 8 week Control and Intervention trials (mean ± SD).

	**Control**		**Within-Group Differences**	**Intervention**		**Within-Group Differences**	**Between-Group Differences**	**Cohen's *d***
	**PRE**	**POST**		**PRE**	**POST**			
**MCA CVR**								
CVR (R^2)	0.89 ± 0.05	0.86 ± 0.07	-0.03 ± 0.08	0.84 ± 0.05	0.87 ± 0.08	0.03 ± 0.09	0.06 ± 0.11	0.74
ABS MCAv ($cm \cdot s^{-1} \cdot mmHg^{-1}$)	3.20 ± 0.89	3.23 ± 1.27	0.03 ± 1.06	3.18 ± 1.09	3.35 ± 0.61	0.17 ± 0.83	0.14 ± 1.11	0.16
REL MCAv ($\% \cdot mmHg^{-1}$)	4.61 ± 0.99	4.69 ± 1.64	0.08 ± 1.23	4.85 ± 1.51	5.14 ± 1.01	0.29 ± 1.25	0.21 ± 1.04	0.18
**CCA CVR**								
ABS Diameter ($cm \cdot mmHg^{-1}$)	0.001 ± 0.001	0.001 ± 0.001	0.000 ± 0.001	0.001 ± 0.001	0.001 ± 0.001	0.000 ± 0.002	0.000 ± 0.002	0.00
REL Diameter ($\% \cdot mmHg^{-1}$)	0.13 ± 0.13	0.13 ± 0.09	0.00 ± 0.14	0.20 ± 0.18	0.13 ± 0.19	-0.07 ± 0.25	-0.07 ± 0.28	0.37
ABS Blood Flow ($ml \cdot min^{-1} \cdot mmHg^{-1}$)	0.26 ± 0.22	0.23 ± 0.21	-0.03 ± 0.22	0.25 ± 0.28	0.17 ± 0.27	-0.08 ± 0.34	-0.05 ± 0.42	0.19
REL Blood Flow ($\% \cdot mmHg^{-1}$)	2.00 ± 1.64	1.87 ± 1.55	-0.13 ± 1.78	2.06 ± 2.20	1.38 ± 1.91	-0.68 ± 2.48	-0.55 ± 3.08	0.27
**NVC**								
ABS Peak PCAv ($\Delta cm \cdot s^{-1}$)	9.8 ± 4.9	10.9 ± 6.3	1.1 ± 3.7	6.8 ± 3.9	6.5 ± 5.4	-0.3 ± 7.4	-1.4 ± 8.0	0.26
REL Peak PCAv (%)	20.6 ± 12.2	22.7 ± 13.7	2.1 ± 8.0	14.7 ± 8.3	15.4 ± 13.7	0.7 ± 17.7	-1.4 ± 18.0	0.11
ABS Peak MCAv ($\Delta cm \cdot s^{-1}$)	6.2 ± 1.2	6.2 ± 3.5	0.0 ± 2.7	4.8 ± 0.6	5.4 ± 1.2	0.6 ± 1.3	0.6 ± 3.5	0.32
REL Peak MCAv (%)	8.9 ± 2.0	9.2 ± 4.2	0.3 ± 3.0	7.5 ± 1.7	8.3 ± 2.5	0.8 ± 2.1	0.5 ± 4.6	0.22
**CA: 5-sec (LF)**								
Phase (degrees)	24.33 ± 5.30	17.87 ± 8.20	-6.46 ± 6.45	24.33 ± 19.96	24.07 ± 6.80	-0.26 ± 16.67	6.19 ± 16.67	0.53
Gain ($cm \cdot s^{-1} \cdot mmHg^{-1}$)	0.87 ± 0.17	0.83 ± 0.12	-0.04 ± 0.12	0.81 ± 0.23	0.98 ± 0.25	0.17 ± 0.22	0.21 ± 0.33	1.25
$Gain_n$ ($\% \cdot mmHg^{-1}$)	1.38 ± 0.25	1.36 ± 0.22	-0.02 ± 0.18	1.30 ± 0.26	1.52 ± 0.37	0.22 ± 0.35	0.24 ± 0.49	0.91
Coherence[#]	0.64 ± 0.11	0.61 ± 0.07	-	0.69 ± 0.12	0.61 ± 0.07	-	-	-
**CA: 10-sec (VLF)**								
Phase (degrees)	43.72 ± 15.11	37.53 ± 10.02	-6.19 ± 7.75	45.33 ± 18.89	46.30 ± 17.94	0.97 ± 17.11	7.16 ± 14.41	0.57
Gain ($cm \cdot s^{-1} \cdot mmHg^{-1}$)	0.74 ± 0.18	0.83 ± 0.22	0.09 ± 0.11	0.72 ± 0.25	0.80 ± 0.21	0.08 ± 0.16	-0.01 ± 0.22	0.08
$Gain_n$ ($\% \cdot mmHg^{-1}$)	1.20 ± 0.30	1.28 ± 0.29	0.08 ± 0.19	1.20 ± 0.28	1.29 ± 0.29	0.09 ± 0.15	0.01 ± 0.27	0.06
Coherence[#]	0.83±0.15	0.86 ± 0.08	-	0.88 ± 0.10	0.89 ± 0.07	-	-	-

[#]Coherence values were used to accept the validity of gain and phase estimates and not compared.

MCAv—middle cerebral artery blood flow velocity; CVR—cerebrovascular carbon dioxide reactivity; ABS—absolute; REL—relative; CCA—common carotid artery; NVC—neurovascular coupling; PCAv—posterior cerebral artery blood flow velocity; CA—cerebral autoregulation; LF—low frequency, VLF-—very low frequency; $Gain_n$—normalised gain.

Table S3. Measures of mood and work productivity at the start (PRE) and following (POST) the 8 week Control and Intervention trials (mean ± SD).

	Control		Within-Group	Intervention		Within-Group	Between-Group	Cohen's *d*
	PRE	POST	Differences	PRE	POST	Differences	Differences	
**Mood**								
Positive Affect	34.9 ± 7.9	35.3 ± 6.1	0.4 ± 7.0	35.5 ± 6.7	35.7 ± 5.5	0.2 ± 6.3	-0.2 ± 9.5	0.04
Negative Affect	15.6 ± 5.2	15.1 ± 3.6	-0.5 ± 5.9	15.7 ± 6.5	15.6 ± 8.2	-0.1 ± 9.4	0.4 ± 12.0	0.05
**HWQ**								
Productivity	6.9 ± 1.9	7.1 ± 1.4	0.2 ± 1.4	6.6 ± 1.9	7.5 ± 1.5	0.9 ± 1.7	0.7 ± 2.1	0.47
Concentration/Focus	4.5 ± 2.5	3.9 ± 2.4	-0.6 ± 2.5	4.6 ± 2.8	3.8 ± 2.3	-0.6 ± 2.2	0.0 ± 4.2	0.00
Impatience/Irritability	3.6 ± 2.3	2.9 ± 2.0	-0.7 ± 2.2	3.7 ± 2.7	2.8 ± 1.5	-0.9 ± 2.5	-0.2 ± 3.5	0.09
Work Satisfaction	7.0 ± 1.8	6.7 ± 2.0	-0.3 ± 1.4	6.7 ± 1.8	7.3 ± 1.5	0.6 ± 1.7	0.9 ± 2.2	0.60
Stress	5.4 ± 2.2	4.4 ± 2.2	-1.0 ± 2.0	5.3 ± 2.9	4.4 ± 2.2	-0.9 ± 3.1	0.1 ± 3.9	0.04
Supervisor Relations	7.0 ± 2.4	6.9 ± 2.3	-0.1 ± 2.9	6.5 ± 2.6	7.0 ± 2.5	0.5 ± 2.5	0.6 ± 4.1	0.23
Non-work Satisfaction	8.2 ± 1.6	8.0 ± 1.5	-0.2 ± 1.1	7.7 ± 2.4	8.2 ± 1.8	0.5 ± 1.9	0.7 ± 2.4	0.47

HWQ—Health and Work Questionnaire.
